# Supplementary material for: Hit screening with multivariate robust outlier detection
Source: PLoS One. 2024 Sep 12;19(9):e0310433. doi: 10.1371/journal.pone.0310433 (PMC11392271; doi:10.1371/journal.pone.0310433)
Supplement: S1 Appendix — Details of the outlier generation method used in the simulation study. (DOCX) [file pone.0310433.s001.docx]

# S1 Appendix

## Outlier generation procedure

For all simulations, inlier data were generated from a *p*-dimensional multivariate normal distribution with mean 0 and covariance matrix $\Sigma$, $MVN\left( \boldsymbol{0}, \boldsymbol{\Sigma} \right)$. For outliers, we first generated $q$ points from a standard *p*-dimensional multivariate normal distribution $\boldsymbol{Y}\sim MVN\left( \mathbf{0}, \boldsymbol{I}_{p} \right)$, where $\boldsymbol{I}_{p}$ is the identity matrix. In the standardized coordinate system, the data are uncorrelated and Mahalanobis distance reduces to the Euclidean distance. By projecting the simulated points onto the surface of a *p*-dimensional sphere with radius $d$, we created outlying observations whose distance to the center of the sphere is $d$. The projected data $\boldsymbol{Z}$ is given by:

$$\boldsymbol{Z}=d\times\frac{\boldsymbol{Y}}{\sqrt{\sum_{j=1}^{p} y_{j}^{2}}}$$

Next, we applied Cholesky transformation to correlate the variables and produce outliers $\boldsymbol{Z}^{*}$ with the desired correlation structure as follow:

$$\boldsymbol{U}=chol\left( \boldsymbol{\Sigma} \right)$$

$$\boldsymbol{Z}^{*}=\boldsymbol{Z}\times\boldsymbol{U}$$

where $chol$ denotes Cholesksy transformation. The outliers $\boldsymbol{Z}^{*}$are placed in random direction around the ellipse.
