# Supplementary material for: Hit screening with multivariate robust outlier detection
Source: PLoS One. 2024 Sep 12;19(9):e0310433. doi: 10.1371/journal.pone.0310433 (PMC11392271; doi:10.1371/journal.pone.0310433)
Supplement: S3 File — Performance of mROUT estimated for 2-, 3- and 96-dimensional simulations with different number of observations. (DOCX) [file pone.0310433.s004.docx]

# S3 File

## Effect of sample size on the performance of mROUT

In this experiment, we investigated the performance of mROUT on simulated data sets (*p* = 2, 3 and 96) with different number of observations (*N* = 20, 40 and 100). Other simulation parameters are the same as those listed in Table 1 in the manuscript except for the levels of outliers, where ε = 1 was not tested because this produced a fraction when *N* < 100. The results are presented in the multi-parts figure below. Simulation outcomes from *N* = 200 were included as benchmark.


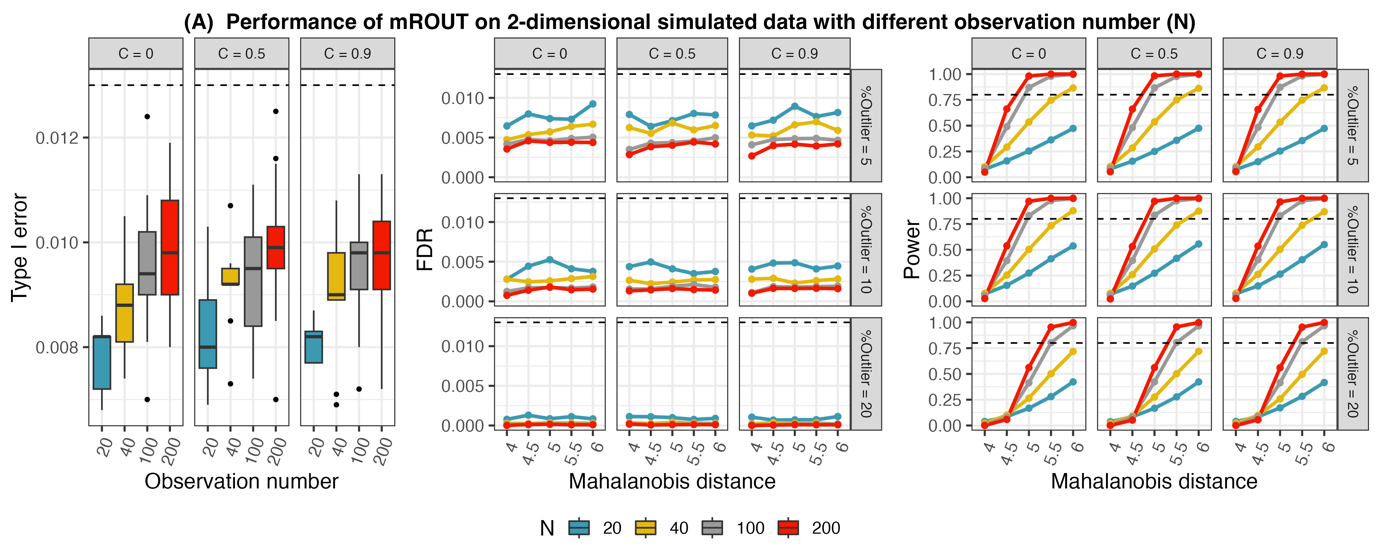


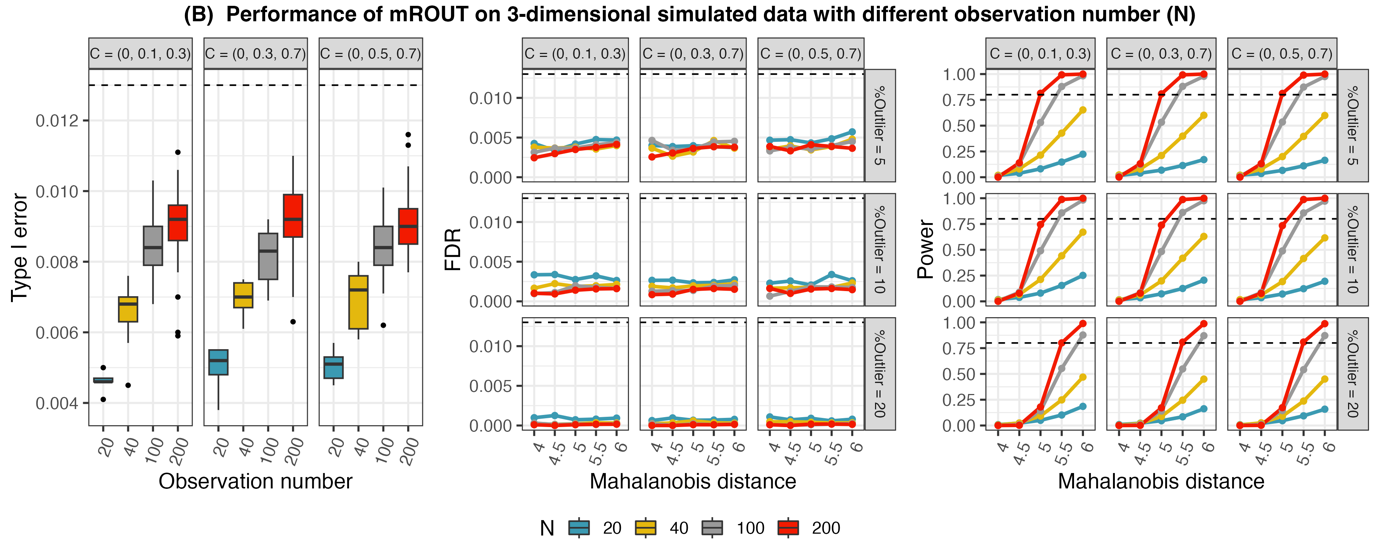


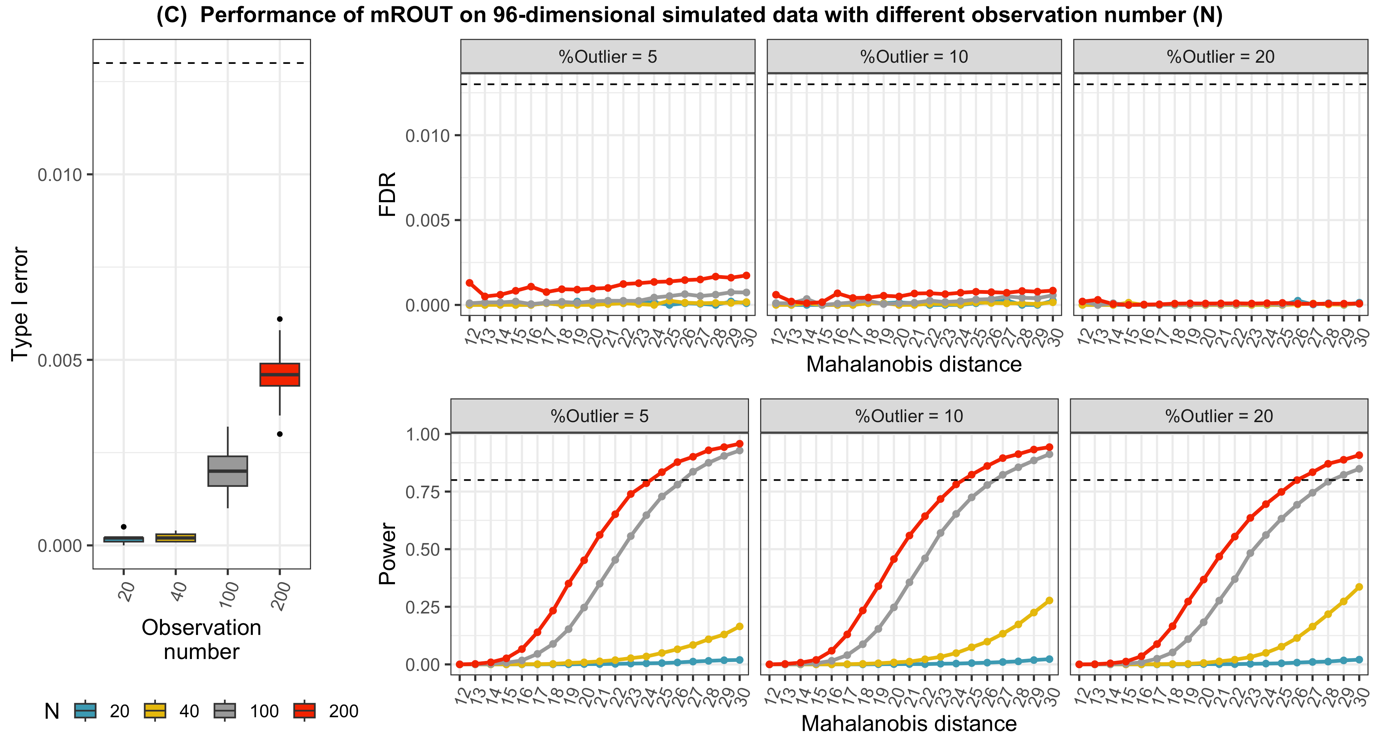


These results demonstrate that Type I error and FDR are consistently maintained at levels below 0.01. However, a noticeable decrease in statistical power is observed when the number of observations is limited. Specifically, the statistical power is substantially lower for *N* = 20 and 40, even in 2- and 3-dimensions. The drop in performance is likely due to insufficient data for accurately estimating the inactive distribution and robust statistics. As *N* becomes larger (>100), statistical power improves markedly, even at 96-dimensional scenario. In summary, statistical power is a function of the number of observations (higher power with larger *N*).
