# Supplementary material for: Hit screening with multivariate robust outlier detection
Source: PLoS One. 2024 Sep 12;19(9):e0310433. doi: 10.1371/journal.pone.0310433 (PMC11392271; doi:10.1371/journal.pone.0310433)
Supplement: S1 Table — Type I error of mROUT and other outlier detection methods estimated for 2-, 3- and 96-dimensional simulations. (DOCX) [file pone.0310433.s006.docx]

**S1 Table.** Type I error estimated for *N* = 200 and nominal Q = 0.01 in the absence of outliers (ε = 0).

| *p* | *C* | mROUT | PcaCov | PcaHubert | PcaGrid | PcaProj | PCOut |
| --- | --- | --- | --- | --- | --- | --- | --- |
| 2 | 0 | 0.010 | 0.011 | 0.011 | 0.009 | 0.009 | 0.117 |
|  | 0.5 | 0.010 | 0.010 | 0.011 | 0.011 | 0.011 | 0.115 |
|  | 0.9 | 0.010 | 0.010 | 0.010 | 0.015 | 0.015 | 0.116 |
| 3 | (0, 0.1, 0.3) | 0.009 | 0.010 | 0.010 | 0.008 | 0.008 | 0.117 |
|  | (0, 0.3, 0.7) | 0.009 | 0.010 | 0.010 | 0.011 | 0.011 | 0.116 |
|  | (0, 0.5, 0.7) | 0.009 | 0.010 | 0.010 | 0.013 | 0.012 | 0.116 |
| 96 | matrix^a^ | 0.005 | - | 0.012 | - | 0.012 | 0.117 |

^a^ A 96 x 96 correlation matrix that mimics the structure in the real data (supplied as Supporting information S2 File).
