# Supplementary material for: Hit screening with multivariate robust outlier detection
Source: PLoS One. 2024 Sep 12;19(9):e0310433. doi: 10.1371/journal.pone.0310433 (PMC11392271; doi:10.1371/journal.pone.0310433)
Supplement: S2 Table — FDR of mROUT and other outlier detection methods estimated for 2- and 3-dimensional simulations. (DOCX) [file pone.0310433.s007.docx]

**S2 Table**. False discovery rate estimated for 2- and 3-dimensional simulations with *N* = 200 and Q = 0.01.

| *p* | ε (%) | *d* | *C1* | | | | | | *C2* | | | | | | *C3* | | | | | |
| --- | --- | --- | --- | --- | --- | --- | --- | --- | --- | --- | --- | --- | --- | --- | --- | --- | --- | --- | --- | --- |
|  |  |  | mROUT | PcaCov | PcaHubert | PcaGrid | PcaProj | PCOut | mROUT | PcaCov | PcaHubert | PcaGrid | PcaProj | PCOut | mROUT | PcaCov | PcaHubert | PcaGrid | PcaProj | PCOut |
| 2 | 1 | 5 | 0.008 | 0.013 | 0.011 | 0.012 | 0.012 | 0.916 | 0.009 | 0.012 | 0.012 | 0.019 | 0.019 | 0.915 | 0.009 | 0.012 | 0.012 | 0.046 | 0.045 | 0.915 |
|  |  | 5.5 | 0.008 | 0.015 | 0.012 | 0.011 | 0.012 | 0.916 | 0.010 | 0.011 | 0.012 | 0.020 | 0.020 | 0.915 | 0.008 | 0.012 | 0.011 | 0.042 | 0.042 | 0.914 |
|  |  | 6 | 0.010 | 0.014 | 0.012 | 0.012 | 0.012 | 0.915 | 0.008 | 0.012 | 0.011 | 0.018 | 0.018 | 0.914 | 0.008 | 0.010 | 0.010 | 0.045 | 0.043 | 0.914 |
|  | 5 | 5 | 0.004 | 0.006 | 0.005 | 0.006 | 0.006 | 0.640 | 0.004 | 0.005 | 0.004 | 0.008 | 0.008 | 0.635 | 0.004 | 0.005 | 0.004 | 0.019 | 0.019 | 0.633 |
|  |  | 5.5 | 0.004 | 0.006 | 0.005 | 0.005 | 0.006 | 0.638 | 0.004 | 0.005 | 0.004 | 0.008 | 0.008 | 0.633 | 0.004 | 0.005 | 0.005 | 0.018 | 0.018 | 0.633 |
|  |  | 6 | 0.004 | 0.006 | 0.005 | 0.005 | 0.005 | 0.639 | 0.004 | 0.005 | 0.004 | 0.008 | 0.008 | 0.634 | 0.004 | 0.005 | 0.004 | 0.018 | 0.018 | 0.632 |
|  | 10 | 5 | 0.002 | 0.002 | 0.002 | 0.002 | 0.002 | 0.394 | 0.002 | 0.002 | 0.002 | 0.004 | 0.004 | 0.389 | 0.002 | 0.002 | 0.002 | 0.009 | 0.008 | 0.388 |
|  |  | 5.5 | 0.001 | 0.002 | 0.002 | 0.002 | 0.002 | 0.393 | 0.001 | 0.002 | 0.002 | 0.004 | 0.004 | 0.388 | 0.002 | 0.002 | 0.002 | 0.008 | 0.008 | 0.388 |
|  |  | 6 | 0.002 | 0.002 | 0.002 | 0.002 | 0.002 | 0.392 | 0.001 | 0.002 | 0.002 | 0.004 | 0.004 | 0.388 | 0.002 | 0.002 | 0.002 | 0.008 | 0.008 | 0.387 |
|  | 20 | 5 | 0.000 | 0.000 | 0.000 | 0.000 | 0.000 | 0.125 | 0.000 | 0.000 | 0.000 | 0.001 | 0.001 | 0.122 | 0.000 | 0.000 | 0.000 | 0.002 | 0.002 | 0.122 |
|  |  | 5.5 | 0.000 | 0.000 | 0.000 | 0.000 | 0.000 | 0.124 | 0.000 | 0.000 | 0.000 | 0.001 | 0.001 | 0.122 | 0.000 | 0.000 | 0.000 | 0.002 | 0.002 | 0.122 |
|  |  | 6 | 0.000 | 0.000 | 0.000 | 0.000 | 0.000 | 0.125 | 0.000 | 0.000 | 0.000 | 0.001 | 0.001 | 0.123 | 0.000 | 0.000 | 0.000 | 0.002 | 0.002 | 0.122 |
| 3 | 1 | 5 | 0.007 | 0.015 | 0.013 | 0.012 | 0.011 | 0.916 | 0.007 | 0.014 | 0.013 | 0.024 | 0.022 | 0.916 | 0.007 | 0.016 | 0.014 | 0.032 | 0.031 | 0.916 |
|  |  | 5.5 | 0.008 | 0.013 | 0.011 | 0.011 | 0.011 | 0.916 | 0.007 | 0.015 | 0.013 | 0.022 | 0.022 | 0.916 | 0.008 | 0.015 | 0.013 | 0.034 | 0.032 | 0.916 |
|  |  | 6 | 0.008 | 0.013 | 0.011 | 0.012 | 0.012 | 0.916 | 0.007 | 0.015 | 0.013 | 0.022 | 0.022 | 0.915 | 0.008 | 0.014 | 0.013 | 0.033 | 0.031 | 0.915 |
|  | 5 | 5 | 0.003 | 0.006 | 0.006 | 0.005 | 0.005 | 0.637 | 0.004 | 0.007 | 0.006 | 0.011 | 0.010 | 0.636 | 0.004 | 0.007 | 0.006 | 0.015 | 0.014 | 0.637 |
|  |  | 5.5 | 0.004 | 0.006 | 0.006 | 0.005 | 0.005 | 0.637 | 0.004 | 0.007 | 0.006 | 0.010 | 0.010 | 0.636 | 0.004 | 0.007 | 0.006 | 0.014 | 0.013 | 0.636 |
|  |  | 6 | 0.004 | 0.006 | 0.005 | 0.005 | 0.005 | 0.637 | 0.004 | 0.007 | 0.006 | 0.009 | 0.009 | 0.636 | 0.004 | 0.007 | 0.006 | 0.012 | 0.012 | 0.635 |
|  | 10 | 5 | 0.001 | 0.003 | 0.002 | 0.002 | 0.002 | 0.395 | 0.002 | 0.003 | 0.002 | 0.005 | 0.004 | 0.393 | 0.002 | 0.004 | 0.003 | 0.007 | 0.006 | 0.393 |
|  |  | 5.5 | 0.002 | 0.003 | 0.002 | 0.002 | 0.002 | 0.394 | 0.002 | 0.004 | 0.003 | 0.005 | 0.004 | 0.391 | 0.002 | 0.003 | 0.003 | 0.006 | 0.005 | 0.392 |
|  |  | 6 | 0.002 | 0.003 | 0.002 | 0.002 | 0.002 | 0.394 | 0.002 | 0.004 | 0.003 | 0.004 | 0.004 | 0.391 | 0.001 | 0.003 | 0.003 | 0.006 | 0.005 | 0.392 |
|  | 20 | 5 | 0.000 | 0.001 | 0.000 | 0.000 | 0.000 | 0.130 | 0.000 | 0.001 | 0.001 | 0.001 | 0.001 | 0.128 | 0.000 | 0.001 | 0.001 | 0.001 | 0.001 | 0.128 |
|  |  | 5.5 | 0.000 | 0.001 | 0.001 | 0.000 | 0.000 | 0.128 | 0.000 | 0.001 | 0.001 | 0.001 | 0.001 | 0.127 | 0.000 | 0.001 | 0.001 | 0.001 | 0.001 | 0.128 |
|  |  | 6 | 0.000 | 0.001 | 0.001 | 0.000 | 0.000 | 0.128 | 0.000 | 0.001 | 0.001 | 0.001 | 0.001 | 0.127 | 0.000 | 0.001 | 0.001 | 0.001 | 0.001 | 0.128 |

For *p* = 2, *C1* = 0, *C2* = 0.5, *C3* = 0.9.

For *p* = 3, *C1* = (0, 0.1, 0.3), *C2* = (0, 0.3, 0.7), *C3* = (0, 0.5, 0.7).
