# Supplementary material for: Hit screening with multivariate robust outlier detection
Source: PLoS One. 2024 Sep 12;19(9):e0310433. doi: 10.1371/journal.pone.0310433 (PMC11392271; doi:10.1371/journal.pone.0310433)
Supplement: S3 Table — Statistical power of mROUT and other outlier detection methods estimated for 2- and 3-dimensional simulations. (DOCX) [file pone.0310433.s008.docx]

**S3 Table**. Statistical power estimated for 2- and 3-dimensional simulations with *N* = 200 and Q = 0.01.

| *p* | ε (%) | *d* | *C1* | | | | | | *C2* | | | | | | *C3* | | | | | |
| --- | --- | --- | --- | --- | --- | --- | --- | --- | --- | --- | --- | --- | --- | --- | --- | --- | --- | --- | --- | --- |
|  |  |  | mROUT | PcaCov | PcaHubert | PcaGrid | PcaProj | PCOut | mROUT | PcaCov | PcaHubert | PcaGrid | PcaProj | PCOut | mROUT | PcaCov | PcaHubert | PcaGrid | PcaProj | PCOut |
| 2 | 1 | 5 | 0.930 | 0.974 | 0.990 | 0.892 | 0.898 | 1 | 0.928 | 0.889 | 0.943 | 0.855 | 0.859 | 1 | 0.927 | 0.388 | 0.395 | 0.779 | 0.783 | 1 |
|  |  | 5.5 | 0.999 | 0.999 | 1 | 0.995 | 0.995 | 1 | 0.999 | 0.917 | 0.961 | 0.968 | 0.971 | 1 | 0.999 | 0.460 | 0.465 | 0.882 | 0.885 | 1 |
|  |  | 6 | 1 | 1 | 1 | 1 | 1 | 1 | 1 | 0.931 | 0.968 | 0.996 | 0.996 | 1 | 1 | 0.533 | 0.536 | 0.934 | 0.936 | 1 |
|  | 5 | 5 | 0.981 | 0.997 | 1 | 0.973 | 0.976 | 1 | 0.983 | 0.914 | 0.957 | 0.927 | 0.932 | 1 | 0.981 | 0.409 | 0.414 | 0.834 | 0.838 | 1 |
|  |  | 5.5 | 1 | 1 | 1 | 1 | 1 | 1 | 1 | 0.936 | 0.967 | 0.989 | 0.991 | 1 | 1 | 0.486 | 0.488 | 0.917 | 0.921 | 1 |
|  |  | 6 | 1 | 1 | 1 | 1 | 1 | 1 | 1 | 0.948 | 0.973 | 0.999 | 0.999 | 1 | 1 | 0.550 | 0.55 | 0.953 | 0.955 | 1 |
|  | 10 | 5 | 0.970 | 0.996 | 0.997 | 0.962 | 0.966 | 1 | 0.970 | 0.928 | 0.956 | 0.916 | 0.918 | 1 | 0.964 | 0.394 | 0.392 | 0.818 | 0.822 | 1 |
|  |  | 5.5 | 0.999 | 1 | 1 | 1 | 1 | 1 | 0.999 | 0.942 | 0.968 | 0.985 | 0.985 | 1 | 0.999 | 0.492 | 0.49 | 0.904 | 0.905 | 1 |
|  |  | 6 | 1 | 1 | 1 | 1 | 1 | 1 | 1 | 0.952 | 0.971 | 0.998 | 0.998 | 1 | 1 | 0.538 | 0.537 | 0.950 | 0.953 | 1 |
|  | 20 | 5 | 0.562 | 0.934 | 0.913 | 0.75 | 0.762 | 1 | 0.561 | 0.878 | 0.875 | 0.752 | 0.759 | 1 | 0.560 | 0.301 | 0.283 | 0.706 | 0.706 | 1 |
|  |  | 5.5 | 0.953 | 1 | 1 | 0.984 | 0.986 | 1 | 0.956 | 0.95 | 0.965 | 0.943 | 0.946 | 1 | 0.954 | 0.437 | 0.430 | 0.851 | 0.854 | 1 |
|  |  | 6 | 0.998 | 1 | 1 | 1 | 1 | 1 | 0.998 | 0.957 | 0.968 | 0.988 | 0.989 | 1 | 0.999 | 0.508 | 0.499 | 0.912 | 0.917 | 1 |
| 3 | 1 | 5 | 0.580 | 0.712 | 0.785 | 0.520 | 0.555 | 1 | 0.591 | 0.437 | 0.450 | 0.582 | 0.614 | 1 | 0.585 | 0.433 | 0.449 | 0.591 | 0.604 | 1 |
|  |  | 5.5 | 0.961 | 0.862 | 0.908 | 0.877 | 0.910 | 1 | 0.965 | 0.588 | 0.592 | 0.844 | 0.872 | 1 | 0.962 | 0.587 | 0.592 | 0.815 | 0.846 | 1 |
|  |  | 6 | 0.999 | 0.891 | 0.921 | 0.988 | 0.993 | 1 | 0.999 | 0.668 | 0.671 | 0.948 | 0.961 | 1 | 1 | 0.676 | 0.678 | 0.915 | 0.94 | 1 |
|  | 5 | 5 | 0.813 | 0.815 | 0.867 | 0.683 | 0.735 | 1 | 0.808 | 0.494 | 0.510 | 0.706 | 0.740 | 1 | 0.812 | 0.503 | 0.503 | 0.691 | 0.720 | 1 |
|  |  | 5.5 | 0.993 | 0.883 | 0.918 | 0.962 | 0.976 | 1 | 0.992 | 0.639 | 0.634 | 0.905 | 0.926 | 1 | 0.990 | 0.637 | 0.645 | 0.878 | 0.899 | 1 |
|  |  | 6 | 1 | 0.915 | 0.936 | 0.998 | 1 | 1 | 1 | 0.709 | 0.709 | 0.973 | 0.98 | 1 | 1 | 0.719 | 0.713 | 0.954 | 0.966 | 1 |
|  | 10 | 5 | 0.745 | 0.821 | 0.853 | 0.643 | 0.699 | 1 | 0.739 | 0.49 | 0.488 | 0.666 | 0.709 | 1 | 0.734 | 0.499 | 0.486 | 0.658 | 0.691 | 1 |
|  |  | 5.5 | 0.988 | 0.893 | 0.920 | 0.95 | 0.968 | 1 | 0.987 | 0.634 | 0.633 | 0.898 | 0.923 | 1 | 0.987 | 0.640 | 0.629 | 0.871 | 0.899 | 1 |
|  |  | 6 | 1 | 0.918 | 0.935 | 0.998 | 0.999 | 1 | 1 | 0.716 | 0.718 | 0.966 | 0.980 | 1 | 1 | 0.712 | 0.707 | 0.946 | 0.963 | 1 |
|  | 20 | 5 | 0.179 | 0.694 | 0.673 | 0.274 | 0.307 | 1 | 0.171 | 0.367 | 0.335 | 0.412 | 0.434 | 1 | 0.173 | 0.379 | 0.346 | 0.438 | 0.459 | 1 |
|  |  | 5.5 | 0.801 | 0.893 | 0.904 | 0.795 | 0.842 | 1 | 0.808 | 0.595 | 0.578 | 0.774 | 0.817 | 1 | 0.809 | 0.584 | 0.585 | 0.755 | 0.797 | 1 |
|  |  | 6 | 0.989 | 0.917 | 0.924 | 0.972 | 0.987 | 1 | 0.987 | 0.676 | 0.671 | 0.919 | 0.942 | 1 | 0.987 | 0.683 | 0.676 | 0.891 | 0.919 | 1 |

For *p* = 2, *C1* = 0, *C2* = 0.5, *C3* = 0.9.

For *p* = 3, *C1* = (0, 0.1, 0.3), *C2* = (0, 0.3, 0.7), *C3* = (0, 0.5, 0.7).
