# Supplementary material for: Hit screening with multivariate robust outlier detection
Source: PLoS One. 2024 Sep 12;19(9):e0310433. doi: 10.1371/journal.pone.0310433 (PMC11392271; doi:10.1371/journal.pone.0310433)
Supplement: Attachment — Submitted filename: review-PONE-D-24-10015.pdf [file pone.0310433.s009.pdf]

Review comments for "Hit screening with multivariate robust outlier detection"

This paper introduces a novel method called mROUT for detecting outliers in multivariate data. The approach combines classical principal analysis for dimension reduction with the one-dimensional robust outlier detection method, ROUT. Through experimentation on both synthetic and real data, the method demonstrates superior performance compared to other state-of-the-art methods.

Overall, this paper is well-written, with accurate main content, although there are some typos. Below are some comments.

- In the dimensional reduction step, it's mentioned that at least 99% of the total variance of X are retained. I'm interested in knowing the value of k in the experiments, particularly for the high-dimensional case (p=96). I'm curious whether the orthogonality of Y is necessary while the dimensional reduction might be unnecessary for mROUT. Could the authors test mROUT with full dimensions? If such comparisons have already been made, it would be beneficial to discuss the necessity of dimensional reduction in mROUT, although the discussion of its necessity in other methods is already included.
- In line 70, provide the full term for MAD.
- In line 74, verify whether "$\sqrt{2}$" is necessary.
- In line 111, the meaning of the notation "-ile" is unclear.
- In line 159, clarify the expression "Even after we modified rPCA-rMD methods to also provide a BH FDR-adjusted p-value ...".
- It appears that both N and MVN are utilized as notations for the multivariate normal distribution in the sections "Multivariate Outlier Detection" and "Materials and Methods," respectively. It would be advisable to use a consistent notation throughout the paper.
- The images in the paper have very low resolution, making them appear blurry.
