# Supplementary material for: Hit screening with multivariate robust outlier detection
Source: PLoS One. 2024 Sep 12;19(9):e0310433. doi: 10.1371/journal.pone.0310433 (PMC11392271; doi:10.1371/journal.pone.0310433)
Supplement: Attachment — Submitted filename: Response to Reviewers.docx [file pone.0310433.s010.docx]

**Response to Reviewers**

**PLOS ONE Manuscript PONE-D-24-10015**

**Hit Screening with Multivariate Robust Outlier Detection**

Dear Dr. Huang,

Thank you for giving us the opportunity to revise our manuscript. We are grateful for the time and consideration that you and the reviewers have given to this manuscript and for the valuable comments. We have provided a detailed response to the reviewers’ comments below (in blue) and incorporated their suggestions where possible. All line numbers in our response refer to the revised manuscript file with tracked changes.

Sincerely,

The authors

***Reviewer #1***

This paper introduces a novel method called mROUT for detecting outliers in multivariate data. The approach combines classical principal analysis for dimension reduction with the one-dimensional robust outlier detection method, ROUT. Through experimentation on both synthetic and real data, the method demonstrates superior performance compared to other state-of-the-art methods.

Overall, this paper is well-written, with accurate main content, although there are some typos. Below are some comments.

Thank you for these supportive comments.

1. In the dimensional reduction step, it’s mentioned that at least 99% of the total variance of X are retained. I’m interested in knowing the value of k in the experiments, particularly for the high-dimensional case (p=96). I’m curious whether the orthogonality of Y is necessary while the dimensional reduction might be unnecessary for mROUT. Could the authors test mROUT with full dimensions? If such comparisons have already been made, it would be beneficial to discuss the necessity of dimensional reduction in mROUT, although the discussion of its necessity in other methods is already included.

For high-dimensional simulation experiments (in our case, *p* = 96), *k* usually takes values between 8−13, with a median of 10 or 11 when we retained at least 99% of the total variance of ***X****.* As mentioned in the manuscript, we have also tested lowering the variance explained to 95%. In this case, the value of *k* reduced to 5 or 6 but the system suffered a drop in performance in terms of an uncontrolled false positive rate.

In terms of the necessity of orthogonality of the reduced score matrix ***Y***, several other authors listed in our references have proposed procedures without orthogonality, suggesting that orthogonality is not necessary. The difficulty with non-orthogonality is the accurate estimation of a *k*-dimensional covariance matrix. When *k* is small (e.g., *k* = 3, 4, 5), we ran simulations and observed that the method exhibited good performance - effectively controlled Type I error and false discovery rate (FDR), while also demonstrating reasonably good statistical power. As the dimension grows, it becomes increasing challenging to obtain a robust and reliable estimate of the covariance matrix, because (i) the number of observations required to accurately estimate the covariance matrix grows exponentially, the available data may be insufficient to reliably estimate the true variance matrix, (ii) multicollinearity can inflate the variance and lead to instability in the estimates, and (iii) high computational burden. In simulations, we found that, for the non-orthogonal case and *k* > 5, the Type I error and FDR were uncontrolled. We have opted to control the complexity in covariance matrix estimation in mROUT by orthogonalizing ***Y*** so that the variance-covariance matrix is diagonal and simpler to compute.

The reviewer raised an interesting question about running mROUT with full dimensions. To address this question with our proposed method, we performed simulation for *p* = 96 without dimensionality reduction using the same parameters described in Table 1 in the manuscript. The PCA step was included because, as explained above, orthogonality is necessary for the mROUT procedure, but *k* was set to 96 in this run. Our data show that while the Type I error and FDR are under control (< 0.01), there is a substantial drop in statistical power (Figure R-1).

We hypothesized that the decline in performance could be attributed to the inclusion of essentially noise columns in the Mahalanobis distance (MD). For instance, if *k* = 10 explains 99% of the variability in the original data matrix, then the MD statistic may sum across an additional 86 units of noise. As illustrated in the density plot of the PCA scores below (Figure R-2), the outliers were predominantly captured by the first few principal components (PCs) and were not prominent in the higher dimensions. Given that all PCs carry equal weight, incorporating irrelevant or redundant PCs in higher dimensions tended to obscure the outlier signal and introduce noise into the MD estimate. In summary, running mROUT with full dimensions is a reasonable suggestion but does not work.

In early development of our proposed method, we did attempt to incorporate the percent variability (and functions of percent variability) of each PCA score column as a weight in the MD statistic. When we tested the system with computer-generated columns of independent, normally-distributed data with known weights and no PCA step, we observed good performance. However, when we tested with multivariate normal data with a PCA step, the weights (percent variability of each column) were approximated. Under this condition, the proposed system veered out of statistical control.

We have included a concise summary of the key findings from this discussion in the “Step 1: Dimensionality reduction” section of the revised manuscript (lines 253-256).

2. In line 70, provide the full term for MAD.

We have changed this to ‘median absolute deviation (MAD)’ in the text.

3. In line 74, verify whether " $\surd$2" is necessary.

The expression $\Phi^{-1}\left( 0.975 \right) \sqrt{2}\sigma$ expresses the Minimum Discriminatory Difference in a theoretical context, where $\Phi^{-1}\left( 0.975 \right)$ is the critical value from the standard normal distribution for a two-tailed test at the 5% significance level. Here, $\sqrt{2}\sigma$ is the combined standard deviation of the difference between two independent samples, each with standard deviation $\sigma$.

Verification of the $\sqrt{2}$factor:

Let $Y_{1}$ and $Y_{2}$ denote two independent normal samples. The variance of the difference between their means is the sum of their variance. For equal variances $\sigma^{2}$, $Var(Y_{1}-Y_{2})=2\sigma^{2}.$Therefore the combined standard deviation of the difference is$\sqrt{2}\sigma$.

4. In line 111, the meaning of the notation "-ile" is unclear.

We have changed this to ‘68.27th percentile’ in the text.

5. In line 159, clarify the expression "Even after we modified rPCA-rMD methods to also provide a BH FDR- adjusted p-value ...".

Apologies for the lack of clarity here. We have added the following text in the revised manuscript (lines 183-188) to clarify this expression:

*To enable a direct comparison of our method and the rPCA-rMD approaches, we adjusted the latter from returning an outlier flag to returning a p-value based on the distance metrics and distributional assumption (chi-square) proposed by the authors, and further corrected the p-value for multiple testing using the BH FDR correction method. That is, the authors provided an outlier flag if* $\hat{d}>\chi^{2}(r)$*, where* $\hat{d}$ *is their test statistic and r is their calculated degrees of freedom.*

6. It appears that both N and MVN are utilized as notations for the multivariate normal distribution in the sections "Multivariate Outlier Detection" and "Materials and Methods," respectively. It would be advisable to use a consistent notation throughout the paper.

Thank you for pointing this out. We have changed the notation in line 134 from *N* to *MVN,* and ensure that *MVN* is used throughout the manuscript when referring to multivariate normal distribution.

7. The images in the paper have very low resolution, making them appear blurry.

We have increased the image resolution and ensure the figures meet PLOS requirements.

***Reviewer #2***

This paper describes a multivariate outlier detection method (mROUT) that combines PCA with robust Mahalanobis distance computation. Some of the key steps include estimating the Cauchy-Lorentz location parameter using maximum likelihood (Levenberg–Marquardt DLS method) and estimating the scatter using the Motulsky-Brown robust standard deviation of residuals (rsdr). The authors have demonstrated in their results the importance of incorporating BH multiplicity-adjustment on the p-value to control the false detection rate (FDR) when outlier detection is conducted via T-squared testing. The motivation and essential details of the proposed algorithm are clearly described. Although the results demonstrate quite convincingly the strengths of the proposal within the current scope of the experiments, further discussion to elucidate the merits and general limitations of the approach should be encouraged. Perhaps the authors can consider re-parameterising/extending some of the experiments and including relevant findings where appropriate. More detailed suggestions are given in particular in Comments 4-8.

Overall, this work represents an original research contribution to the field of phenotypic screening / biostatistics. Specifically, the authors have developed an FDR-calibrated multivariate outlier detection approach that may facilitate more effective identification of potential targeted treatments. In practical terms, it may lead to discovery of compounds for the treatment of complex diseases, and gaining insights into novel pathways or mechanisms. The reviewer is intrigued to see some of the phenotypic changes following treatment and the use of convolutional neural network, self-supervised contrastive learning in a binary classification task to represent well images and differentiate between inactive and active controls; then applying outlier detection to features extracted from the penultimate layer of a CNN. The results (e.g. line 399-406) show tremendous promise. The reviewer hopes this can be scaled up and adopted more widely.

We thank the reviewer for these constructive comments.

List of Comments

**[Comment 1]** Introduction (L39-40)

1a) Regarding normally distributed inactives [as observed in multi-dimensional cell painting or morphological profiling] - how often is this a valid assumption?

The assumption of normally distributed inactives in multi-dimensional cell painting or morphological profiling data is not universally valid. The multi-task convolutional neural network (CNN) approach that we used in this work produces feature data that are Gaussian-like – certainly enough to be a useful approximation. For profiles generated with other image analysis software, the data may exhibit skewed or other non-normal characteristics. The recommended practice is to transform feature values with mathematical operations, such that the feature values approximate a normal distribution, mean centered and have comparable standard deviation to facilitate downstream analysis (see review by Caicedo et al. 2017). Therefore, we think our proposed approach will be generally applicable for many cases after appropriate transformation or preprocessing of the image features.

We have clarified this in the revised manuscript (lines 217-231) and included the Caicedo’s review article as reference:

Caicedo, J., Cooper, S., Heigwer, F. et al. Data-analysis strategies for image-based cell profiling. Nat Methods 14, 849–863 (2017). <https://doi.org/10.1038/nmeth.4397>

1b) What methods are available for handling situations where the Gaussian assumption is violated? PCA seems like a reasonable choice when there is no preferential direction (all variables are valued equally) or non-linearity observed in the point distribution. When this is not true, have the authors considered using local neighbourhood embedding [1] or kernel techniques [2] to handle non-Gaussian data for the inactives? Any thoughts regarding the suitability of manifold learning methods?

As the reviewer implies, our method will suffer in the presence of non-Gaussian data. The columns of the principal components from a PCA will not be univariate normally distributed if the original data matrix is not approximately multivariate normal. It is a common practice to perform statistical tests or visual inspections (e.g., Q-Q plot) to assess data distribution. Additionally, empirical methods like bootstrapping and permutation tests can also be used to confirm distributional assumptions. If the data were found to be non-Gaussian, simple data transformation like log, square-root, or other Box-Cox power transformations can make the data more normally distributed.

We agree with the reviewer that PCA may not be well-suited for non-linear and non-Gaussian data. In the presence of non-linear relationships, non-linear dimensionality reduction techniques such as t-distributed Stochastic Neighbor Embedding (t-SNE) or Isomap may be more appropriate. When dealing with non-Gaussian data, methods such as Independent Component Analysis (ICA) or kernel PCA, which can handle non-Gaussian distributions, may be more suitable.

We started our research journey using t-SNE and UMAP, where we attempted to formulate a distance metric from these transformations. However, we ultimately transitioned to PCA for several reasons. Firstly, we wanted our method to use a metric that calculates a distance from each compound to inactives. A reliance on the multivariate normal distribution in the PC space was necessary to use the F-distribution for the MD statistic. Secondly, we found that many dimension reduction methods did not result in a multivariate ellipsoid, at times producing banana shapes in 2-dimensional space, making it difficult to define the distribution of inactives. Furthermore, most non-PCA dimension reduction methods do not provide a percentage of information explained by using *k* columns. Thus, we could not know if we captured enough essence of the original data through the dimension-reduction procedure (e.g., t-SNE software often provides only 2-3 score columns). Finally, when we decided that orthogonality was important to our procedure, we found that other non-PCA dimension reduction methods yielded correlated matrices.

While we have not explored all of the alternative techniques, we have considered a more holistic approach by incorporating a variational autoencoder (VAE) into our image feature extraction workflow. The VAE serves to enforce the distribution of the latent space alongside the reconstruction loss through the inclusion of the Kullback-Leibler divergence term in the overall loss function. This regularization encourages the VAE to learn a latent space adhering to a specific distribution - in our case, a standard normal distribution - while simultaneously optimizing for precise data reconstruction. Given the necessity for orthogonality and explained variance, we believe the VAE approach may be the best route for applying our method to strongly non-Gaussian data, as may be obtained from other morphological profiling pipelines, such as CellProfiler.

Several of the concepts from our reply to this question were added to the revised manuscript (lines 168-175, 563-571).

1c) A statistical comparison of outlier detection efficacy - with and without these embedding, manifold learning or kernel techniques - may be considered as part of future work.

[1] Jiaqi Xue, Bih Zhang, Qianyao Qiang. Local Linear Embedding with Adaptive Neighbors, Pattern Recognition, vol. 136, article 109205, Apr 2023. [DOI: [https://doi.org/10.1016/j.patcog.2022.109205](https://url.de.m.mimecastprotect.com/s/Sq0qC46Aq2C6nPLnXUjILBW?domain=doi.org)
[2] P. Rudra, R. Baster, E. Hsieh, D. Ghosh. Compositional data analysis using kernels in mass cytometry data, Bioinformatics Advances, vol. 2., issue 1., 2022. DOI: [https://doi.org/10.1093/bioadv/vbac003](https://url.de.m.mimecastprotect.com/s/OKiKC57BrMFRz8qzBtlMuAS?domain=doi.org)

Thank you for the suggestion and references. We added some text in the introduction and conclusion sections to refer to other dimension reduction algorithms. These methods fall outside the scope of our work. We have considered using Bayesian multivariate kernel techniques for outlier detection/hit screening, especially Gaussian and Dirichlet processes, that split the data into two or more “clusters”. Each data point would then be evaluated against its probability of belonging to one group or another. We found it difficult to decide on the number of groups, instead opting to define only the inactives group, which led to our eventual decision to forego the use of multivariate kernel techniques.

**[Comment 2]** Multivariate outlier detection (L158-161)

The authors observed that "even after we modified rPCA-rMD methods to also provide a BH FDR-adjusted p-value..., we found that our method had better Type I error control, better FDR control, and/or higher statistical power to declare H1 in (1) compared with other methods." Can you offer some explanation as to why these other techniques produced inferior results despite the p-value FDR adjustment?

These rPCA-rMD procedures are based on Minimum Covariance Determinant (MCD), projection pursuit (PP) or a combination of both. Our 2- and 3-dimensional simulation results show that the performance of these approaches is influenced by the correlation structure of the data, in particular when the variables are highly correlated.

In high-dimensional data with a complex correlation structure, MCD may struggle to accurately estimate the covariance matrix - the presence of strong correlations can distort the estimation of the covariance matrix, potentially resulting in an underestimation of the scatter matrix and consequently misidentifying outliers.

In the case of PP, identifying meaningful projections that effectively capture the outliers becomes challenging - the presence of correlated variables can lead to the projection space being dominated by certain dimensions, potentially masking the presence of outliers in other dimensions.

We think that these limitations may have contributed to the inferior results observed in our simulations.

**[Comment 3]** mROUT algorithm, step 2

3a) (L208-209) Was the Levenberg–Marquardt nonlinear regression algorithm used to estimate the Cauchy-Lorentz location parameter? If so, please mention and include a reference (e.g. [4]). What are its competitive advantages?

[4] H.P. Gavin, The Levenberg-Marquardt algorithm for nonlinear least squares curve-fitting problems, 2024. DOI: [https://people.duke.edu/~hpgavin/ExperimentalSystems/lm.pdf](https://url.de.m.mimecastprotect.com/s/p_WnC6WDvMuMgkQg5S99hgR?domain=people.duke.edu)

3b) Can you provide some information about the optimizer used to maximise the likelihood. In terms of practicality, any particular reason for using the Nelder and Mead (1965) method, or not using quasi-Newton and L-BFGS-B.

Response to 3a and 3b: We used the optim() function in R with the default “Nelder-Mead” option to estimate the Cauchy location parameter. The Nelder-Mead method is a heuristic optimization technique that belongs to the class of direct search methods (citation is given below). We did not try quasi-Newton and L-BFGS-B because we found that the Nelder-Mead method is generally robust and effective for our problem here.

The reference for the Nelder-Mead algorithm is:

Nelder, J. A. and Mead, R. (1965).  A simplex algorithm for function minimization. *The Computer Journal*, Volume 7, Issue 4, January 1965, Pages 308–313.  doi: 10.1093/comjnl/7.4.308 (URL:<https://doi.org/10.1093/comjnl/7.4.308>).

We added some of these comments to the Materials and methods section (lines 278-282).

3c) Did you encounter any difficulty estimating the Cauchy parameters using real data.

3d) Have you implemented any checks to ensure the estimates are sensible, i.e., detect anomalies or reject poor estimates. Do you envisage any situation where the maximum likelihood technique might fail *silently* when deployed in a large scale setting?

Response to 3c and 3d: We did not encounter any issues with the convergence parameters in both real and simulated data. Given that we fit the likelihood function to the centered and scaled PCA scores, the optimization task becomes particularly straightforward as we are aiming to estimate zero as the center. We verified the convergence in each of our simulated runs. While there may be scenarios in which the maximum likelihood technique could fail, identifying anomalies in the convergence of this algorithm is beyond the scope of this paper.

**[Comment 4]** Simulation (L237)

An important question is whether the performance of the proposed method would hold for fewer observations (e.g. N between 20 and 50)?

Recommendation 1 - Where possible, the experiments should be repeated for different N, and graphs plotted to observe any changes in the results.

Thank you for the suggestion. We have extended our experiments to investigate the performance of mROUT on simulated data sets (*p* = 2, 3 and 96) with different number of observations (*N* = 20, 40 and 100). Other simulation parameters are the same as those listed in Table 1 of the revised manuscript except for the levels of outliers, where ε = 1 was not tested because this produced a fraction when *N* < 100.

The results presented below include simulation outcomes for *N* = 200 as benchmark (Figure R-3). These data demonstrate that Type I error and FDR are consistently maintained at levels below 0.01. However, a noticeable decrease in statistical power is observed when the number of observations is limited. Specifically, the statistical power is substantially lower for *N* = 20 and 40, even in 2- and 3-dimensions. The drop in performance is likely due to insufficient data for accurately estimating the inactive distribution and robust statistics. As *N* becomes larger (>100), statistical power improves markedly, even at 96-dimensional scenario.

These results have been incorporated into the revised manuscript (lines 450-460) and in Supporting information (S3 File).

**Figure R-3**

**[Comment 5]** Application to real data (L402-403) "Using a significance threshold Q=0.01, mROUT identified 12 gene KOs as hits. All ACs and LCs were detected and none of the ICs were declared as hits"

5a) This is encouraging. It would be helpful to provide some guidance on the selection of Q, if we want to view this as a parameter tuning exercise.

Recommendation 2 - Visualise in a graph how statistical power (sensitivity) and FDR vary with Q. This will help readers outside a clinical setting to appreciate the type 1 and type 2 errors trade-off (often the F1 score is skewed depending on the application, sometimes higher recall is preferred over precision).

We used the same Q value as recommended by Motulsky and Brown in the original ROUT paper. By setting Q = 0.01, we are looking to limit “hits” to observations that fall beyond the 99^th^ percentile of the inactive distribution. We agree with the reviewer that it would be beneficial to assess the performance of mROUT across different Q values.

We have carried out additional experiments to evaluate the effects of varying Q within the range of 0.001 to 1. The simulations were conducted with *p* = 96, *N* = 200, incorporating different levels of contamination at 1%, 5%, 10% and 20%. Mahalanobis distance (MD) values of 15, 20, 25 and 30 with reasonable discovery rate were used. In each scenario, 10,000 Monte Carlo runs were performed to estimate the true positive rate (TPR) and false positive rate (FPR).

The results are visualised as receiver operating characteristic (ROC) curves (Figure R-4). The trajectory of these curves shows that mROUT effectively constrains the false positive rate (FPR) across the range of tested Q values. While TPR (sensitivity) remains low when MD = 15, it rises to over 75% when MD exceeds 25, without compromising the FPR. In all the scenarios tested, increasing Q from 0.01 to 0.05 lead to an improvement in the TPR, but has minimal impact on the FPR. These findings demonstrate that mROUT is well-suited for applications where prioritizing higher recall is advantageous. It also alleviates concerns about incurring an excessively high false positive rate when using a higher Q value.

We have included these results and discussion in the revised manuscript (lines 461-488).

General Questions - not tied to a specific section

**[Comment 6]** In chemometrics, data can have dimensions in the thousands. Is there evidence that the proposed technique would work equally well for this case (where the number of variables p >> 96)? Would it make sense to use projection pursuit initially to retain the most critical information (by whatever criteria) and reduce the number of dimensions to something more manageable?

Our current focus is on applying the proposed technique to data with dimensions in the hundreds. Our simulations have revealed that using too few PCA columns leads to a loss of control over Type I error. As a result, we have chosen to retain 99% of the variability to mitigate this issue. While we anticipate that this technique will remain effective in much higher dimensions, we acknowledge the potential for an increased risk of false positives.

We have not explored projection pursuit (PP) and would like to thank the reviewer for prompting us to consider this technique as an initial step for dimension reduction in our procedure. PP and other techniques discussed in Comment 1(b) above could be investigated as part of our future efforts to extend the application of mROUT to high-dimensional non-Gaussian data.

The concern is that PCA explains data variance using eigenvectors aligned with directions of maximum variation. This is optimal for linear decomposition. However, the reliability of this procedure depends on the proportion and magnitude of the outliers (relative to the inactives). Second, the Euclidean metric is only meaningful if important differences are reflected by large distances as measured by the L2 norm. Are the authors aware of any situation where nonlinear dimensionality reduction would be of benefit, where manifold learning would be needed to preserve small differences or latent structures of biological significance?

We did examine UMAP, which is a nonlinear dimensionality reduction method. Because we wished to use parametric testing, we chose the L2 norm through Mahalanobis distance. The UMAP method fell short for two reasons. Firstly, its dimension reduction step could lead to a banana shape in 2-dimensional space. Such a shape has an unknown distribution and so the test statistic distribution would be unknown. Secondly, UMAP did not provide a percentage of variability explained. Due to the lack of information regarding the UMAP transformation’s content, we were not confident to use it. We think that the Mahalanobis distance approach would not be appropriate for nonlinear dimensionality reduction. We appreciate the suggestion as it has prompted us to explore new directions for future research.

**[Comment 7]** What are some of the known limitations of the proposed method? For example, for tiny data sets, how would it behave if applied in another problem domain where the number of measurements N is small (e.g. N between 20-50, rather than > 200)? This is related to Comment 4. Can this be explored through Monte Carlo simulation, by randomly drawing say {25, 50, 100} samples from the complete set of measurements (which acts as ground truth) to emulate such effect? The reviewer is thinking how this would affect the multiplicity-adjusted p values and hypothesis testing outcomes.

The proposed method is a generalization of T-test for hit screening and, as such, shares similar limitations. If the original data conforms to a multivariate normal distribution, the theory underlying our technique will hold, albeit with reduced statistical power as *N* decreases, as discussed in Comment 4. However, if the original data distribution deviates from multivariate normal, our proposed method may suffer from model misspecification, potentially yielding misleading conclusions.

**[Comment 8]** How would the proposed method behave given perfect data (with no outliers) in high dimensions (let's say the retained PCA components k far exceeds 11), and garbage data of a psuedo-random nature with no trends at all. Does the current design raise a flag, to perhaps alert scientists that the data is unexpected or perhaps some error has been introduced during the experiments?

The PCA step will eliminate data columns that lack any discernible signal. If the reviewer’s concern relates to random data with no signal, we have addressed this through experiments demonstrating control of Type I error. If the reviewer is inquiring about the method’s capability to detect violations of model assumptions, we believe this aspect falls beyond the scope of the present study and may warrant exploration in future research.

***Reviewer #3***

A new multivariate robust outlier identification technique is presented in this paper. Despite the appealing idea and promising methodology, the discussions, conclusions, and result interpretation have some significant shortcomings. Additionally, there are major improvements in writing and presentation required. Here are some main significant problems (from my point of view).

1. The literature review and proper citations are missing. It is necessary to update the References. The references are extremely old, with several dating back to before 2010, and there is just one reference from 2021.

While our focus was primarily on PCA-based outlier detection methods, we acknowledge the importance of incorporating a comprehensive literature review and appropriate citations. We will add references for more recent papers with different methodologies, based on this reviewer’s suggestions from question 2.

2. The existing techniques for detecting outliers multivariate data are not well reviewed. Numerous recent papers have presented robust techniques for the identification of outliers. A comparative analysis of the offered new approach in this study and existing techniques in recent papers is significantly needed.

Here are examples of few recent papers:

- Vishwakarma et al., 2021. A hybrid feedforward neural network algorithm for detecting outliers in non-stationary multivariate time series. Expert Systems with Applications 184:115545
- Touny et al., 2024. Scalable fuzzy multivariate outliers identification towards big data applications. Applied Soft Computing 155: 111444
- Vishwakarma et al., 2023. An automated robust algorithm for clustering multivariate data. Journal of Computational and Applied Mathematics 429: 115219
- Hilal et al., 2022. FinanciaL Fraud: A Review Of Anomaly Detection Techniques And Recent Advances. Expert Systems with Applications 193: 116429

We thank the reviewer for the references, we will include them in the manuscript. While none of the suggested methods conform with the type of data we propose to analyze, their inclusion will enable us to contrast our method with existing techniques. Our data is derived from a single snapshot in time, which differs from the time series data context for the neural network approach described in Vishwakarma et al*.* (2021). The fuzzy clustering multivariate outlier detection introduced by Touny et al. (2024) and Vishwakarma et al*.* (2023) requires more than one cluster, while our data is conceptualized as originating from a single cluster with a few outliers. Finally, the techniques presented in Hilal et al. (2022) are tailored for much larger datasets, entailing the training of a model, which does not align with the nature of our analysis.

3. The main article must include tables that present some performance measures to compare the new method and the existing techniques.

Thank you for the suggestion. We have added a table to show the performance comparison between mROUT and existing techniques in high dimensional simulation study (see Table 2 in the revised manuscript). Given the complexity of the remaining results (involving various combinations of factor levels), we believe that the visual representations of Type I error, false discovery rate, and statistical power effectively convey the necessary information to the reader.

4. The organization and presentation of the results, along with a lack of clear interpretation are major challenges. There should be some discussion on the theoretical arguments for the new method's superiority.

In response to comments from this and other reviewers, we have undertaken an expansion and re-parameterization of several experiments to provide a more comprehensive exploration of the strengths and weaknesses inherent in the proposed approach. The relevant findings have been incorporated into the revised manuscript, accompanied by discussions that stem from these adjustments.

5. There is a need to enhance the resolution of the figures.

We have increased the image resolution and ensure the figures meet PLOS requirements.
