# Supplementary material for: Hit screening with multivariate robust outlier detection
Source: PLoS One. 2024 Sep 12;19(9):e0310433. doi: 10.1371/journal.pone.0310433 (PMC11392271; doi:10.1371/journal.pone.0310433)
Supplement: Attachment — Submitted filename: Response to Reviewers.docx [file pone.0310433.s011.docx]

**Response to Reviewers**

**PLOS ONE Manuscript PONE-D-24-10015R1**

**Hit Screening with Multivariate Robust Outlier Detection**

Dear Dr. Huang,

We deeply appreciate the valuable feedback provided on the revised manuscript. The thorough review process and the meticulous attention to detail demonstrated by the reviewers have been instrumental in refining and strengthening the quality of our work.

We have carefully addressed the minor comments from this round of review, as outlined in our responses below (in blue). The manuscript has been further adjusted accordingly. We hope that the revised manuscript meets your expectations and will be considered for publication in PLOS ONE following this round of review. Thank you once again for your time and consideration.

Sincerely,

The authors

***Reviewer #1***

Thanks for revising the manuscript.

Before it is published, I have three tiny comments:

1. A period is needed at the end of equations between the lines, especially equations in lines 241 and 249. For equations in lines 128 and 264, it would be better to add a comma at the end of the equations. For equation (1), I also suggest adding a comma or a semicolon at the end of the first line and a period at the end of the second line.

The suggested changes have been incorporated into the revised manuscript.

2. When mentioning "Supporting information," consider using "supporting information" or "Supporting Information." Also, since S1_File.pdf is the code, why not upload the raw code file directly?

Thank you for the suggestions. We have adjusted the in-text citations to “supporting information” and submit the raw code file directly as S1_File.R.

3. The images in Figure 2 are still unclear. Although they might meet the PLOS requirements, I suggest using vector format for all images in the manuscript. If you prefer to use the current images, please enlarge the images in Figure 2 as they are hard to see carefully because they are small and some lines are close.

We thank the reviewer for pointing this out. All figures were prepared at 600 dpi resolution in TIFF format and evaluated using the imaging review tool, PACE (https://pacev2.apexcovantage.com/), provided by PLOS to ensure the figures meet publication standards and legible when rendered in a simulated journal page.

***Reviewer #2***

The authors are thanked for responding judiciously to my previous comments. By extending the experiments, and incorporating relevant changes in the revised paper, the authors have acted on both my recommendations and strengthened this manuscript considerably. One area of improvement is informing readers of the strengths and limitations of the proposed methodology.

In relation to changes in performance as the number of observations (N) is varied, the authors demonstrated that Type I error and FDR are consistently maintained at levels below 0.01; however statistical power is noticeably reduced when N is roughly below 40 (see Supporting Info S3). In relation to the significance threshold (Q), the author discovered that Q may be pushed from 0.01 to 0.05 to increase the True Positive Rate with negligible impact on the False Positive Rate, in the context of their experiments (see L425-444 and ROC curves in Fig.4). These additional insights elevate the quality of this contribution. Such guidance is invaluable as it assists researchers/practitioners in making informed choices during experimental design. The comments/discussions relating to non-Gaussian distributed features, convergence, cautionary remarks and techniques that specifically deal with nonlinearity (L197-205, L254-258 and L516-524, resp.) are appreciated.

The issues identified in the previous review have been satisfactorily addressed. On this basis, this reviewer recommends acceptance of this article for publication in PLOS.

Thank you for the thoughtful feedback. The reviewer’s insightful and constructive suggestions have been vital in shaping the improved version of our manuscript.

A question is left below for the authors to ponder. This question (C-2) is, in my opinion, inconsequential for the publication decision. It is more about reflection and academic curiosity.

Minor comments

**C-1** [Author comments, Figure R-1] The vertical axis appears to be mislabelled. Should it be the error of omission "1 - Power"?

The label of the vertical axis is correct - it is indeed “Power” that we intended to show in Figure R-1. The low values on the vertical axis were due to a steep decline in statistical power when running mROUT without dimensionality reduction. Without dimensionality reduction, it appears that the vast majority of extra columns only contribute noise, dramatically attenuating the mROUT statistic’s ability to detect outliers. This implies that the dimension reduction step is critical.

**C-2** [Author's response to Reviewer 2, Figure R-3] In the top-left plot for 2D simulated data, it is interesting to observe the Type 1 error increases with N. This trend is the opposite of FDR(N) which decreases as N increases.

a) This reviewer appreciates the Type 1 error is well controlled irrespective of N (remains below 0.01), but why are there fewer False Positives with fewer observations (for smaller N) and more False Positives with more observations (for larger N). If the Type I error is not under control, the inference would be "limit your observations" which seems a little counterintuitive.

We thank the reviewer for this insightful question. Type 1 error is technically the power to detect an outlier when there is no true outlier in the system. With a larger number of observations (N), the statistical model becomes more accurate and precise, thereby enhancing its power to detect deviations from the inactives. Given that the Type 1 error rate remains below the pre-defined significance level (Q = 0.01), we think the marginal increase in Type 1 error with N is consistent with a more sensitive outlier detection process rather than any fundamental issue. In a properly conducted hypothesis test with independent observations and correct model assumptions, the Type 1 error should not be a function of sample size (with notable exceptions, such as binomial testing). In our case, we are making a few necessary approximations, so we ended up seeing this pattern in Type 1 error with sample size.

b) If we consider a minimum volume ellipsoid (MVE) estimator, one would expect the location and scatter matrix (center and covariance structure of the ellipsoid) will be optimized to cover the inliers in the dataset, and the determinant of the positive definite symmetric matrix will be minimised in the process, subject to this cover constraint. In view of the finding in the Type 1 error graph, can we expect the volume to become more compact as an increasing function of N?

Thank you for mentioning MVE. We explored minimum covariance determinant (MCD) during the course of this work. Our understanding suggests that both MVE and MCD aim to achieve similar objectives by identifying a compact ellipsoid to contain the data. MCD minimizes the determinant of the variance matrix and MVE minimizes the volume of the ellipsoid. According to Rocke and Woodruff (1996), MCD is more efficient compared to MVE. Regardless of efficiency, we did not explore the MVE estimator, mainly because the robust outlier procedures we evaluated were based on MCD. We expect similar operational characteristics from MCD and MVE. Potentially, one algorithm or the other will result in more data points falling outside the ellipsoid and subsequently flagged as outliers as N increases. In general, with more data, we can expect the MVE to more accurately capture the overall structure of the data and resulting in a more compact volume. However, the exact behaviour depends on the specific characteristics and distribution of the data.

The reference of the Rocke and Woodruff’s paper is:

Rocke, D. M., & Woodruff, D. L. (1996). Identification of Outliers in Multivariate Data. Journal of the American Statistical Association, 91(435), 1047–1061. https://doi.org/10.1080/01621459.1996.10476975

***Reviewer #3***

The authors have adequately addressed my comments raised in a previous round of review and I feel that the revised version is much better and can be accepted.

Thank you. We are grateful for the reviewer’s suggestions and comments, which have been invaluable in strengthening our manuscript.
